# Effects of liraglutide on metabolic syndrome in WBN/Kob diabetic fatty rats supplemented with a high‐fat diet

**DOI:** 10.1002/ame2.12106

**Published:** 2020-03-16

**Authors:** Noriyuki Kaji, Yoshiichi Takagi, Satomi Matsuda, Anna Takahashi, Sakurako Fujio, Fumitoshi Asai

**Affiliations:** ^1^ Laboratory of Veterinary Pharmacology School of Veterinary Medicine Azabu University Kanagawa Japan

**Keywords:** diabetes mellitus, liraglutide, metabolic syndrome, models, animal, obesity

## Abstract

**Background:**

Liraglutide, a GLP‐1 receptor agonist, has recently been used to treat metabolic syndrome (MS) because of its anti‐diabetic and anti‐obesity effects. We have previously shown that Wistar Bonn Kobori diabetic and fatty (WBN/Kob‐Lepr*^fa^*, WBKDF) rats fed a high‐fat diet (HFD) developed MS including marked obesity, hyperglycemia, and dyslipidemia. To obtain further information on WBKDF‐HFD rats as a severe MS model, we performed a pharmacological investigation into the anti‐MS effects of liraglutide in this model.

**Methods:**

Seven‐week‐old male WBKDF‐HFD rats were allocated to three groups (N = 8 in each group): a vehicle group, a low‐dose liraglutide group, and a high‐dose liraglutide group. They received subcutaneous injections of either saline or liraglutide at doses of 75 or 300 μg/kg body weight once daily for 4 weeks.

**Results:**

Results showed that liraglutide treatment reduced body weight gain and food intake in a dose‐dependent manner. The marked hyperglycemia and the glucose tolerance were also significantly ameliorated in the liraglutide‐treated groups. Moreover, liraglutide also reduced the plasma triglyceride concentration and liver fat accumulation.

**Conclusions:**

The present study demonstrated that liraglutide could significantly alleviate MS in WBKDF‐HFD rats, and the reaction to liraglutide is similar to human patients with MS. WBKDF‐HFD rats are therefore considered to be a useful model for research on severe human MS.

## 1 INTRODUCTION

Metabolic syndrome (MS) is an epidemic that represents a major health problem worldwide and is characterized by central obesity. The presence of abnormal metabolic parameters, such as central obesity, dyslipidemia, and hypertension, further increase the risk of cardiovascular death in MS.^1^ The high prevalence of MS is probably due to the contemporary prevalence of obesity, unhealthy diet, and sedentary lifestyle.^2^ Weight loss of 5%‐10% has been shown to reduce complications related to obesity and improve the quality of life.^3^ However, weight loss is difficult to maintain with lifestyle intervention alone.^4^

The increased number of MS patients worldwide has stimulated the development of experimental models that mimic the characteristics of human MS,^5^ in attempts to understand the biochemical, physiological, and pathological alterations involved in the development and maintenance of excess body fat and MS.^6^ Several genetic models mimic many of the features of MS occurring in humans such as obese Zucker rats, obese spontaneously hypertensive rats (Koletsky rats) and stroke‐prone SHR‐fatty (*fa/fa*) rats.^7^ However, animal models that develop characteristics of MS not only with genetic manipulation, but also through consumption of specific nutritionally unbalanced diets are increasingly important^8^ for use in simulating the most common causes of human MS. The Wistar Bonn Kobori (WBN/Kob) diabetic fatty (WBKDF) rat is a congenic strain developed by introduction of the *fa* allele of the Zucker fatty rat into the parental WBN/Kob (lean) rat genome.^9^ WBKDF rats have been shown to have severe obesity, insulin resistance, and hyperlipidemia, all of which lead to the development of type 2 diabetes mellitus (T2DM).^9^, ^10^ Epidemiological studies have found that consumption of high‐fat diets (HFDs; ≥30% of energy from fat) is correlated with high rates of being overweight, central obesity and MS.^11^ To develop an animal model mimicking the structural and functional features of MS in people with T2DM, HFD feeding is commonly used in experimental animals. Our previous study demonstrated that 4‐week feeding of a HFD caused diet‐induced obesity and aggravated hyperlipidemia and hyperglycemia in WBKDF rats.^12^

Liraglutide, a glucagon‐like peptide‐1 (GLP‐1) receptor agonist, is approved for the treatment of T2DM at doses up to 1.8 mg once daily^13^ and for weight loss at up to 3.0 mg once daily.^14^ It exerts several glycemic and nonglycemic effects, including the regulation of glucose levels by stimulating glucose‐dependent insulin secretion and the suppression of glucagon secretion.^15^ Liraglutide also shows beneficial effects on obese individuals with prediabetes to reduce the risk for progression to T2DM.^16^

The aim of this study was to validate and demonstrate the potential of WBKDF‐HFD rats as an experimental model of human severe MS. We investigated whether liraglutide treatment resulted in body weight reduction and amelioration of glucose and lipid metabolism in WBKDF‐HFD rats.

## 2 MATERIALS AND METHODS

### 2.1 Test animals and growth conditions

Male WBKDF rats obtained from Japan SLC (Shizuoka, Japan) were housed under standard laboratory conditions (20‐26°C, 50%‐70% humidity) and maintained on a 12/12‐hours light/dark schedule (lights on at 7:00 am) with free access to a sterile HFD (45% kcal from fat, catalog number: 58V8, PMI Nutrition International) and water for 12 weeks. Daily food intake and weekly gains in body weight were routinely recorded throughout the experimental period. All animal experimental procedures were carried out in accordance with the principles of laboratory animal care and approved by the Ethics Committee of Azabu University (Kanagawa, Japan).

### 2.2 Research protocol

HFD feeding of WBKDF rats started at 6 weeks of age (n = 24) and continued for 5 weeks. At 7 weeks of age, WBKDF‐HFD rats were allocated to three groups (eight rats each): a vehicle group, a low‐dose liraglutide group, and a high‐dose liraglutide group. They received subcutaneous injections of either saline or liraglutide (Victoza; Novo Nordisk Pharma) at doses of 75 or 300 μg/kg body weight once daily for 4 weeks. The doses of liraglutide were determined according to results from previous studies.^17^, ^18^ Blood samples were taken once weekly from the tail vein of nonfasting and conscious rats and plasma was used for glucose measurement. Daily food intake was measured by calculating the changes in the diet weight over 24 hours and the averaged food intake for a week is presented.

### 2.3 Intravenous glucose tolerance test

An intravenous glucose tolerance test was performed after 4 weeks of saline or liraglutide treatment and fasting for 18 hours, according to previous studies.^17^, ^19^ Animals were anesthetized using isoflurane (Mylan), and a glucose solution (20 w/v%; Otsuka Pharmaceutical) was injected into the jugular vein at a dose of 0.5 g/kg body weight. Blood samples (0.2 mL) were collected from the jugular vein before and 2, 5, 10, and 20 minutes after the glucose injection. After centrifugation, plasma was collected and used for glucose measurement. Glucose elimination rates were calculated as the slope of the natural logarithm of glucose concentration versus time from 5 to 20 minutes.

### 2.4 Measurements of fat content

After being sacrificed by exsanguination under anesthesia, the epididymal and mesenteric fat pads and the liver were collected and weighed.

### 2.5 Measurement of plasma glucose and lipids

Plasma glucose was measured by an enzymatic colorimetric test kit (Glucose CII‐Test Wako; Wako Pure Chemicals). Biochemistry analysis was performed on an automatic analyzer (JCA‐BM 2250; JEOL Ltd.) using commercial kits with the following parameters: triglycerides (TG), total cholesterol (T‐CHO), and phospholipid (PL).

### 2.6 Histopathological examination of the liver

A histopathological examination of the liver was performed. Briefly, livers obtained from the rats were fixed in 10% neutral buffered formalin (pH 7.4) overnight and then embedded in paraffin. Paraffin‐embedded tissues were sliced, fixed onto treated microscope slides, stained with hematoxylin and eosin (H&E) and Oil Red O. The histopathological examination was performed in a blinded manner.

### 2.7 Statistical analysis

Statistical analyses were performed using GraphPad Prism version 6.0 (GraphPad Software Inc) by one‐way analysis of variance, followed by a post‐hoc Dunnett test. Data are expressed as means ± standard error of the mean (SEM) of the indicated number of determinations. A *P* < .05 was considered significant.

## 3 RESULTS

### 3.1 Effects of liraglutide on obesity in WBKDF‐HFD rats

**FIGURE 1:**
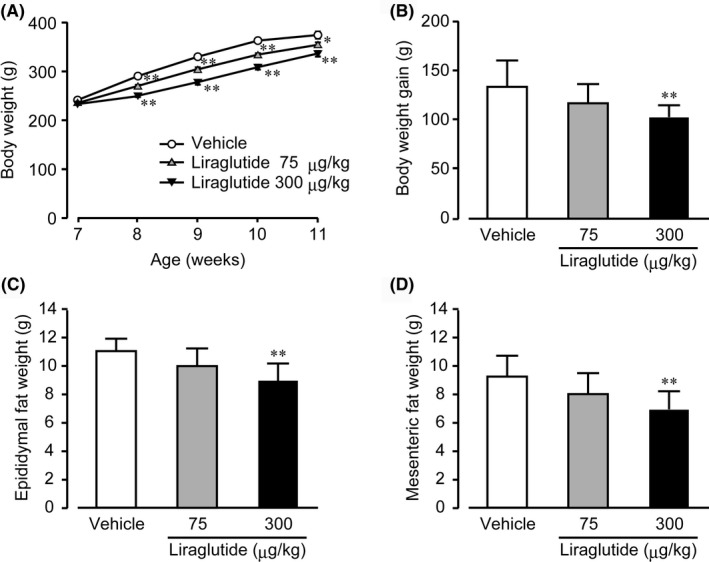
Effects of liraglutide on obesity. (A) Body weight, (B) body weight gain, (C) epididymal, and (D) mesenteric fat weight in high‐fat diet‐supplemented WBN/Kob‐Lepr*^fa^* rats were measured. These rats were treated with a vehicle, low‐dose (75 μg/kg), or high‐dose (300 μg/kg) liraglutide for 4 wk. Data are expressed as mean ± SE (n = 8). *P < .05; **P < .01 versus control rats

No intergroup difference in body weight was observed among the three groups of WBKDF‐HFD rats at 7 weeks of age. During the experimental period, body weights in all groups increased with age (Figure 1A). However, liraglutide treatment caused a significant and dose‐dependent reduction in body weight gain compared to the vehicle group (Figure 1B). The weights of epididymal fat (Figure 1C) and mesenteric fat (Figure 1D) were significantly and dose‐dependently reduced in liraglutide‐treated rats compared to vehicle rats.

### 3.2 Effects of liraglutide on food intake in WBKDF‐HFD rats

**FIGURE 2:**
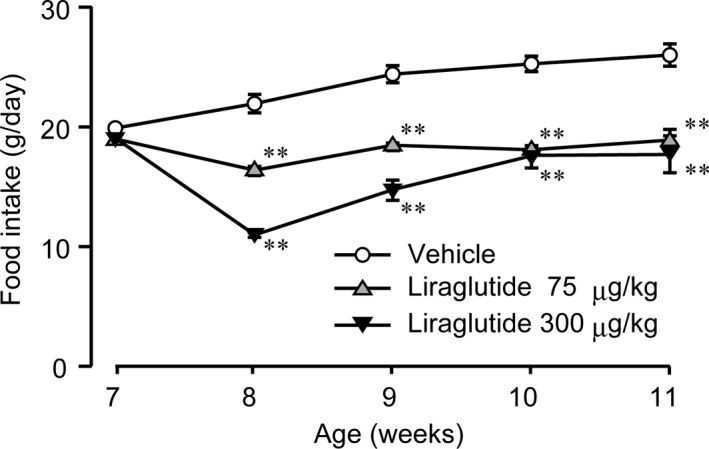
Effects of liraglutide on daily food intake. Daily food intake in high‐fat diet‐supplemented WBN/Kob‐Lepr*^fa^* rats was measured. These rats were treated with a vehicle, low‐dose (75 μg/kg), or high‐dose (300 μg/kg) liraglutide for 4 weeks. Data are expressed as mean ± SE (n = 8). **P < .01 versus control rats

No intergroup difference in the averaged daily food intake was observed among the three groups of WBKDF‐HFD rats at 7 weeks of age. The average daily food intake was significantly decreased in liraglutide‐treated groups compared to the vehicle group (Figure 2). A dose‐dependent reduction in food intake was observed up to 2 weeks after the treatment (at 9 weeks of age).

### 3.3 Effects of liraglutide on hyperglycemia

**FIGURE 3:**
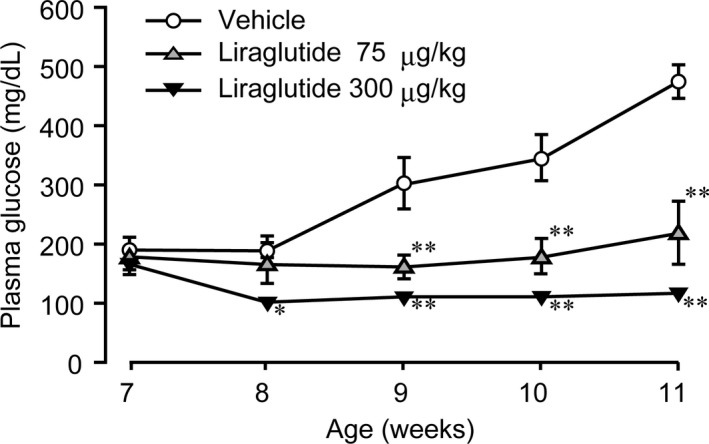
Effects of liraglutide on plasma glucose level. The plasma glucose level in high‐fat diet‐supplemented WBN/Kob‐Lepr*^fa^* rats was measured. These rats were treated with a vehicle, low‐dose (75 μg/kg), or high‐dose (300 μg/kg) liraglutide for 4 wk. Data are expressed as mean ± SE (n = 8). **P < .01 versus control rats

No intergroup difference in the plasma glucose levels was observed among the three groups of WBKDF‐HFD rats at 7 weeks of age. The plasma glucose level of the vehicle group gradually increased with age and it was above 300 mg/dL at 9 weeks of age, which can be considered as the onset of hyperglycemia. In contrast, liraglutide treatment significantly and dose‐dependently inhibited the occurrence of hyperglycemia (Figure 3).

### 3.4 Effect of liraglutide on glucose tolerance

**FIGURE 4:**
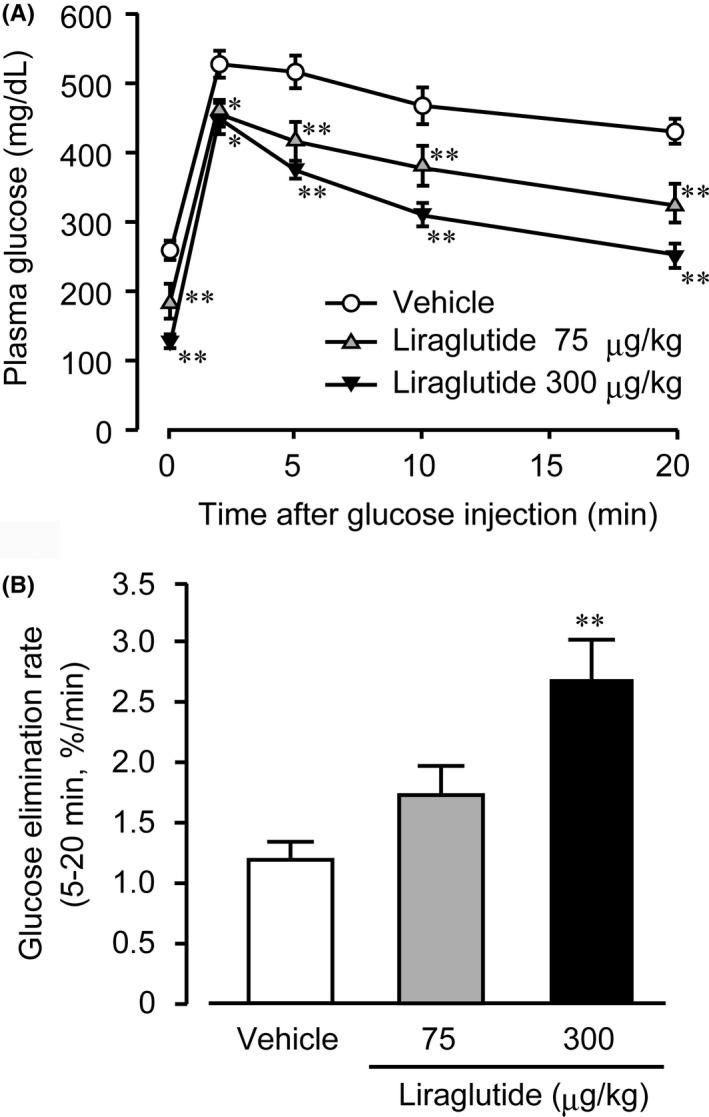
Effects of liraglutide on glucose tolerance. An intravenous glucose tolerance test was performed on high‐fat diet‐supplemented WBN/Kob‐Lepr*^fa^* rats after 4‐wk treatment with a vehicle, low‐dose (75 μg/kg), or high‐dose (300 μg/kg) liraglutide. Changes in plasma glucose after the glucose injection at a dose of 0.5 g/kg body weight were measured (A) and glucose elimination rates between 5 and 20 min were calculated from these values (B). Data are expressed as mean ± SE (n = 8). *P < .05, **P < .01 versus control rats

The fasting plasma glucose level was significantly and dose‐dependently lower in the liraglutide‐treated groups of WBKDF‐HFD rats than that of the vehicle group (Figure 4A, 0 minute). The plasma glucose level after the glucose injection was also significantly decreased in the liraglutide‐treated groups at all measurement points (Figure 4A, 20‐20 minutes). The glucose elimination rate was significantly increased in the high‐dose group, indicating improved glucose tolerance (Figure 4B). The glucose elimination rate of the low dose group was also slightly increased compared to that of the vehicle group.

### 3.5 Effect of liraglutide on plasma lipids

After the 4‐week treatment, plasma levels of T‐Cho and PL were dose‐dependently decreased by liraglutide treatment, with no significance difference between the liraglutide groups and the vehicle group (Table 1). In contrast, liraglutide significantly decreased plasma TG levels in a dose‐dependent manner.

**TABLE 1:** Effects of a vehicle, low‐dose (75 μg/kg), or high‐dose (300 μg/kg) liraglutide on plasma lipids in high‐fat diet‐supplemented WBN/Kob‐Lepr*^fa^* rats

Variable	Control	Liraglutide	
		75 μg/kg	300 μg/kg
Plasma T‐Cho (mg/dL)	174 ± 10.1	167 ± 7.00	157 ± 5.00
Plasma PL (mg/dL)	351 ± 22.4	342 ± 15.4	311 ± 10.5
Plasma TG (mg/dL)	774 ± 99.9	475 ± 89.0*	230 ± 26.9**

Note

Data are expressed as the mean ± SEM (n = 8).

Abbreviations: PL, phospholipid; T‐Cho, total cholesterol; TG, triglycerides.

Significant differences from the control group are indicated (*P < .05, **P < .01, Dunnett's test).

### 3.6 Effect of liraglutide on liver fat accumulation

**FIGURE 5:**
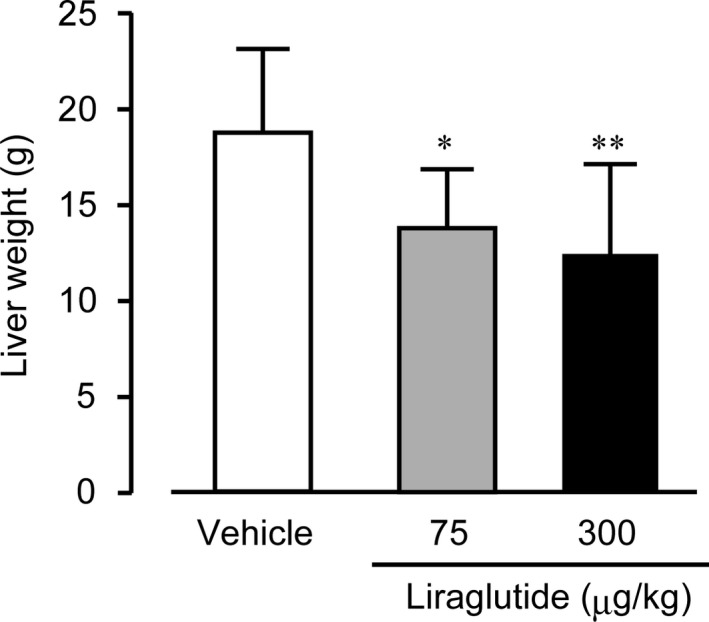
Effects of liraglutide on liver weight. The liver weight of high‐fat diet‐supplemented WBN/Kob‐Lepr*^fa^* rats was measured. These rats were treated with a vehicle, low‐dose (75 μg/kg), or high‐dose (300 μg/kg) liraglutide for 4 wk. Data are expressed as mean ± SE (n = 8). *P < .05; **P < .01 versus control rats

**FIGURE 6:**
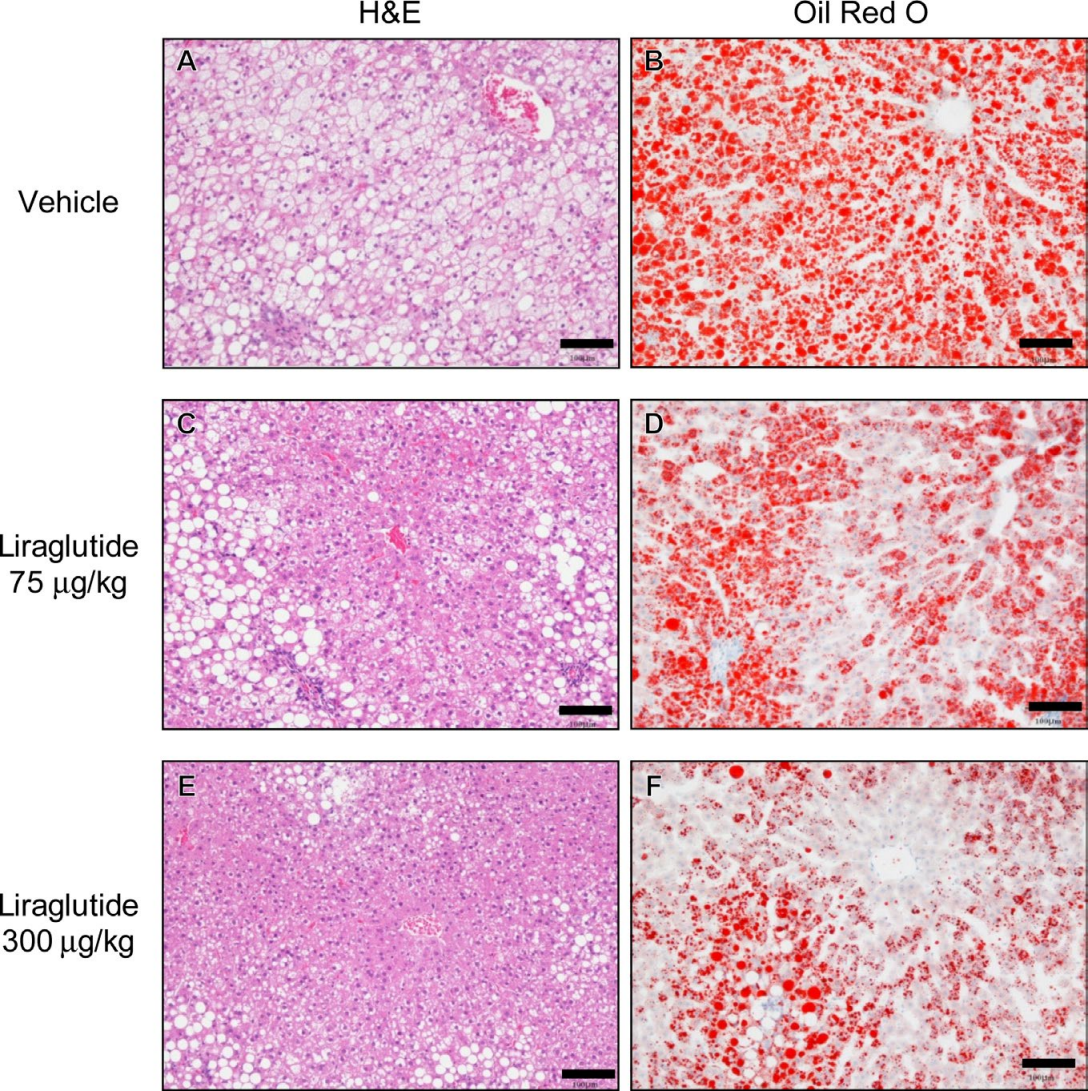
Representative H&E staining and Oil Red O staining of liver sections. H&E staining (A, C, E) and Oil Red O staining (B, D, F) of liver sections from high‐fat diet‐supplemented WBN/Kob‐Lepr*^fa^* rats at the end of the experimental period was performed (Scale bar = 100 µm). These rats were treated with a vehicle (A and B), low‐dose (75 μg/kg, C and D), or high‐dose (300 μg/kg, E and F) liraglutide for 4 wk

Liraglutide treatment reduced liver weight in WBKDF‐HFD rats (Figure 5). Hepatic sections of the control group showed diffuse fatty changes in all the hepatocytes associated with hepatocellular hypertrophy. The degree of fatty changes was dose‐dependently attenuated in the liraglutide‐treated groups (Figure 6).

## 4 DISCUSSION

The availability of useful animal models reflecting human obesity syndrome is crucial in the search for new pharmacological compounds to treat obesity. In the previous study, we developed rat models of human MS by feeding HFD or a fructose‐rich diet (FRD) to WBKDF rats, and showed that WBKDF‐HFD rats developed obesity, dyslipidemia, and especially severe hyperglycemia compared to WBKDF‐FRD rats.^12^ To obtain further information on the model as a potential experimental animal model of severe MS, we performed a pharmacological investigation on the effects of liraglutide, which is a currently approved GLP‐1 agonist for the treatment of T2DM and obesity.^20^, ^21^ WBKDF‐HFD rats developed pathological conditions of MS including central obesity, hyperglycemia, dyslipidemia, as well as hepatosteatosis, closely mimicking human MS. In this study, liraglutide treatment for 4 weeks dose‐dependent decreased body weight gain, fat pad tissue weight, plasma glucose and TG. It also improved glucose tolerance and hepatosteatosis in WBKDF‐HFD rats.

Pre‐diabetic WBKDF rats fed a standard diet are obese and this may be associated with hyperphagia due to the *fa* gene.^9^ HFD causes diet‐induced obesity in humans and various experimental animals, including WBKDF rats.^12^ Accordingly, the obesity that develops in WBKDF‐HFD rats includes genetic obesity and HFD‐induced obesity components. Liraglutide treatment reduced body weight and fat pad weight in WBKDF‐HFD rats in a dose‐dependent manner. These results are consistent with a previous study in humans that showed a body weight reduction by liraglutide was associated with a reduction in visceral fat weight.^22^ These anti‐obesity effects of liraglutide were probably due to its multiple action sites involving the brain and gastrointestinal tract, with the primary action related to an increase in satiety,^23^ consistent with the reduced food intake observed in the liraglutide groups in the present study.

Currently, incretin‐based therapies, which include GLP‐1 receptor agonists and dipeptidyl peptidase‐4 inhibitors, are among the most widely used therapies for human T2DM. As a result, the significance of the incretin effect for the maintenance of glucose homeostasis is clearly established in human T2DM patients,^24^ and also in experimental animals with T2DM including Zucker fatty diabetic rats^25^ and WBKDF rats.^17^ Similarly, in the present study, we found a significant reduction in plasma glucose levels and glucose tolerance aggravated by HFD supplementation in WBKDF rats. These anti‐hyperglycemic effects of liraglutide were probably due to its insulinotropic action.^17^, ^18^, ^25^

The most common lipoprotein abnormalities in patients with diabetes include hypertriglyceridemia associated with increased concentrations of TG‐rich lipoproteins and their remnants.^26^, ^27^ In the present study, liraglutide treatment reduced plasma levels of lipids, especially TG, in WBKDF‐HFD rats, consistent with reported results that GLP‐1 agonists ameliorated dyslipidemia in a human study.^28^ Several mechanisms have been implicated in the lipid‐modifying effects of the incretin hormones and the incretin‐based therapies including decreased dietary fat absorption, a direct decrease in the production and secretion of chylomicrons in the circulation, a decrease in the circulating levels of free fatty acids, and an insulin‐mediated decrease in the intestinal and hepatic production of TG‐rich lipoproteins.^29^ In contrast, these effects of GLP‐1 on lipid metabolism may be independent of the GLP‐1 receptor, since they were not abolished by the administration of a GLP‐1R blocker.^30^

Nonalcoholic fatty liver disease (NAFLD), one of the most common liver diseases, is associated with MS and its components, including obesity, insulin resistance and T2DM.^31^ Simple steatosis is the early stage of NAFLD. The present study showed that WBKDF‐HFD rats exhibited histopathological changes indicating hepatic steatosis. Liraglutide treatment ameliorated these pathological changes, consistent with previous studies on experimental animals fed HFD.^32^, ^33^ The beneficial effects of GLP‐1 receptor agonists on NAFLD in humans have also been demonstrated in several studies.^34^, ^35^ The decrease in liver weight in liraglutide‐treated animals may be caused by the amelioration of fatty liver. Although the mechanisms of GLP‐1 receptor agonists on NAFLD have not yet been clearly explained, clinical and experimental studies have suggested that GLP‐1 therapies may directly exert actions on the liver through activation of GLP‐1 receptors in hepatocytes, resulting in the regulation of gene expression associated with insulin secretion and lipid metabolism.^36^, ^37^

The current study showed that WBKDF‐HFD rats share a number of important characteristics, especially marked hyperglycemia, compared with WBKDF‐FRD rats,^12^ with human MS patients. In addition, this study clearly showed a strong response of WBKDF‐HFD rats to liraglutide, which is used clinically to treat human MS. Our data highlight the usefulness of WBKDF‐HFD rats for a screening of compounds affecting food intake, body weight, glucose homeostasis, dyslipidemia and NAFLD. The WBKDF‐HFD rat model represents a basic structure that could serve as a future foundation for more mechanistic anti‐MS translational models.

This study has several limitations. Energy expenditure was not measured in the current study. In addition, plasma levels of insulin and intra‐hepatic lipid levels were not measured. Further studies are needed to elucidate the mechanism of liraglutide's effects on obesity and hepatosteatosis in WBKDF‐HFD rats.

In summary, the current study demonstrated that the marked visceral obesity, hyperglycemia, dyslipidemia, and hepatic steatosis that developed in WBKDF‐HFD rats were ameliorated by the subcutaneous administration of liraglutide. It also suggested the potential of WBKDF‐HFD rats as an experimental model on severe human MS.

## CONFLICT OF INTEREST

None.

## AUTHOR CONTRIBUTIONS

NK conceived and wrote the original draft of the manuscript. YT and FA revised the manuscript. SM, AT, and SF performed the experiments and analysis. All authors critically read and contributed to the manuscript, and approved the final version.
